# Supplementary material for: A Creative and Movement-Based Blended Intervention for Children in Outpatient Residential Care: A Mixed-Method, Multi-Center, Single-Arm Feasibility Trial
Source: Children (Basel). 2023 Jan 24;10(2):207. doi: 10.3390/children10020207 (PMC9954900; doi:10.3390/children10020207)
Supplement: Supplementary file 1 [file children-10-00207-s001.zip › Supplementary material_BirnkammerCalvano/S1.pdf]

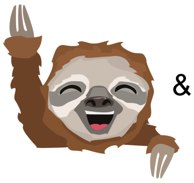

& Action, Datum: ________
Dein Code: ___________

Prä-Testung der Kinder

Hallo,

bevor ihr Teil unseres Projektes & Action werdet, möchten wir euch zuerst etwas besser kennenlernen. Dafür haben wir einige Fragen vorbereitet. Die Fragen sind entweder zum Ankreuzen oder zum selbst beantworten. Dazu haben wir unter diesen Frage etwas Platz gelassen, damit ihr eure Antworten dort aufschreiben könnt. Lasst euch soviel Zeit, wie ihr braucht. Falls ihr Fragen habt, sprecht uns gerne an und wir helfen euch! Solltet ihr bei einer Frage auch mit unserer Hilfe auf keine Antwort kommen, dann lasst die Frage einfach frei und macht mit der Nächsten weiter!
Bitte beantworte die Fragen ehrlich. Keine Angst! Die Antworten sind alle anonym, das heißt, wir wissen nicht, wer sie beantwortet hat.

Viel Spaß bei der Beantwortung der Fragen!

Euer Team von &Action

I. Angaben zum Kind/ Jugendlichen

**Frage 1: Wie alt bist du?**
Schreibe hier dein Alter hin: ______________________________

**Frage 2: Welches Geschlecht hast du? Bitte ankreuzen.**	
	Weiblich
	Männlich
	Anderes (z.B. nicht-binär): ________________

**Frage 3: Welches der folgenden Hobbys hast du ausprobiert? Bitte ankreuzen**	
	In der Natur sein
	Yoga
	Tanzen/Theater/Singen
	Malen/Basteln

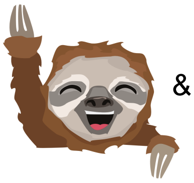

& Action, Datum: _________
Dein Code: ____________

Frage 4: Welches der folgenden Hobbys machst du gerne? Bitte ankreuzen	
	In der Natur sein
	Yoga
	Tanzen/Theater/Singen
	Malen/Basteln

II. Angaben zur Mediennutzung

Frage 5: Welche der nachfolgenden Geräte nutzt du am häufigsten? Nummeriere sie der Häufigkeit nach von 1 (am häufigsten) bis 4 (am wenigsten).	
Nr. __	Handy
Nr. __	Computer/Laptop
Nr. __	Tablet
Nr. __	Spielekonsole

Frage 6: Wie viel Zeit verbringst du durchschnittlich pro Tag am Smartphone? Bitte ankreuzen.	
	weniger als 30 Minuten
	30 - 60 Minuten
	1 - 2 Stunden
	3 - 4 Stunden
	mehr als 5 Stunden

**Frage 7: Wie sicher fühlst du dich im Umgang mit Apps? Bitte ankreuzen.**	
	sehr sicher
	sicher
	teilweise sicher
	nicht sicher
	gar nicht sicher

**Frage 8: Welche Apps benutzt du besonders gerne?**

**Frage 9: Wie hat sich dein Medienkonsum (= die Zeit am Laptop, Smartphone oder Tablet) seit der Pandemie verändert? Bitte ankreuzen.**	
	deutlich mehr
	mehr
	gleich geblieben
	weniger
	deutlich weniger

**Frage 10: Wie findest du deine Menge an Medienkonsum? Bitte ankreuzen.**	
	sehr gut
	gut
	mittel
	schlecht
	sehr schlecht

Herzlichen Dank für die Beantwortung der Fragen! Wir wünschen dir viel Spaß bei dem Projekt! Dein Team von &Action
