# Supplementary material for: A Creative and Movement-Based Blended Intervention for Children in Outpatient Residential Care: A Mixed-Method, Multi-Center, Single-Arm Feasibility Trial
Source: Children (Basel). 2023 Jan 24;10(2):207. doi: 10.3390/children10020207 (PMC9954900; doi:10.3390/children10020207)
Supplement: Supplementary file 1 [file children-10-00207-s001.zip › Supplementary material_BirnkammerCalvano/S2.pdf]

& Action, Datum: ________
Dein Code: ___________

Interviewleitfaden & Action ist ein Projekt der YIN YOUNG & YOU gUG
(haftungsbeschränkt) Ausgründung der Freien Universität Berlin

Projektlaufzeit: 01.01.2022 - 31.08.2022

YIN YOUNG & YOU
Mondori App

Titel der Studie: & Action! The effectiveness of a creative blended care intervention for children in residential care: Results of a single arm, multicenter pilot study

Post Testung der Kinder und Jugendlichen

Susanne Birnkammer, Wissenschaftliche Mitarbeiterin am Fachbereich Erziehungswissenschaften und Psychologie, Arbeitsbereich Klinische Kinder- und Jugendpsychologie und -psychotherapie, Habelschwerdter Allee 45, 14195 Berlin, E-Mail: susanne.birnkammer@fu-berlin.de

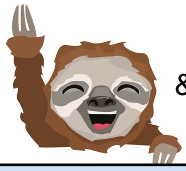

I. Einleitung Post- Testung der Kinder

➜ **Gesprächsbeginn:**

Hallo,
wir hoffen, euch hat unser Projekt gefallen! Nachdem ihr nun in der Welt von Mondori unterwegs gewesen seid, möchten wir euch noch einmal ein paar Fragen dazu stellen. Falls ihr eine Frage nicht versteht, dann sprecht das gerne an und wir helfen euch gerne weiter!

Viel Spaß bei der Beantwortung der Fragen!
Euer Team von & Action!

II. Angaben zum Wohlbefinden

➜ Bevor wir mit dem eigentlichen Interview beginnen, haben wir noch einige Fragen zu deinem Wohlbefinden. ….

**Frage 1: Wie fühlst du dich jetzt, nachdem du Mondori ausprobieren konntest? Kannst du dabei Gefühle benennen?**

III. Angaben zur App

➜ Nachdem wir jetzt erfahren haben wie du dich fühlst, möchten wir dir noch paar Fragen zur App stellen. Ist das für dich in Ordnung?

**Frage 2: Fandest du das Design der App altersgemäß/ansprechend für dein Alter?**

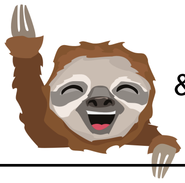

Frage 3: Nenne uns 3 Dinge, die dir an der App besonders gut gefallen haben?

Frage 4: Nenne uns 3 Dinge, die dir an der App überhaupt nicht gefallen haben?

**Frage 5: Wie hat dir das Design von Mondori gefallen? Vergebe eine Schulnote. Bitte ankreuzen.**	
Bewertungshinweis 1-6	
a	1
b	2
c	3
d	4
e	5
f	6

**Frage 6: Würdest du die App Mondori gerne weiter verwenden? Bitte ankreuzen.**	
a	Ja, ich möchte Mondori gerne weiterhin benutzen!
b	Nein, ich möchte Mondori nicht mehr benutzen!

**Frage 7: Würdest du die App Mondori deinen Bekannten weiterempfehlen? Bitte ankreuzen.**	
a	Ja, ich werde die Mondori App weiterempfehlen!
b	Nein, ich werde die Mondori App nicht weiterempfehlen!

**Frage 8: Wenn nein, warum möchtest du Mondori nicht mehr benutzen? Bitte ankreuzen.**	
a	Langweilig
b	Kein Interesse
c	Zu wenig Input (Inseln)
d	Kein Grund
e	Kein Mobiltelefon zur Verfügung
f	Keine Zeit

IV. Angaben zum Projekt

**Frage 9: Welche Aktivität des Projekts in der Einrichtung hat dir besonders gut gefallen? Falls du dich nicht entscheiden kannst, kannst du auch mehrere ankreuzen.**	
	Theater
	Natur
	Kunst
	Yoga

**Frage 10: Was fandest du im Projekt interessanter - die gemeinsamen Übungen oder die App? Bitte ankreuzen.**	
	Aktivitäten
	App
	beides gleich

**Frage 11: Hättest du dir eine längere Laufzeit des Projektes (App mit 12 Inseln, mehrere Wochen mit gemeinsamen Übungen) gewünscht? Bitte ankreuzen.**	
	Ja
	Nein
	Vielleicht, wenn folgendes anders wäre: ___________________________________

**Frage 12: Was hat dir insgesamt am Projekt am besten gefallen?**

V. Gesprächsabschluss

➜ Damit wären wir am Ende unseres Interviews. Wir bedanken uns herzlichst für die Beantwortung der Fragen und dass du Mondori ausgetestet hast! Wir hoffen, es hat dir gefallen! Wir freuen uns, wenn du Mondori auch in der Zukunft weiterhin verwendest!
Ihr Team von &Action!
