# Supplementary material for: A Creative and Movement-Based Blended Intervention for Children in Outpatient Residential Care: A Mixed-Method, Multi-Center, Single-Arm Feasibility Trial
Source: Children (Basel). 2023 Jan 24;10(2):207. doi: 10.3390/children10020207 (PMC9954900; doi:10.3390/children10020207)
Supplement: Supplementary file 1 [file children-10-00207-s001.zip › Supplementary material_BirnkammerCalvano/S3.pdf]

& Action, Datum: ________
Code: ___________

Liebe Mitglieder der Institutionen,

bevor wir Sie Teil des & Action Projektes werden, möchten wir gerne vorab ein paar Fragen an Sie richten, um die Kinder und Jugendlichen besser kennenzulernen. Wir stehen für Fragen jederzeit zur Verfügung. Bitte lassen Sie sich in der Beantwortung soviel Zeit, wie nötig und antworten Sie so ausführlich und ehrlich wie möglich. Die Antworten sind natürlich anonymisiert und nicht mehr auf Sie oder das Kind rückführbar.

Ihr Team von &Action

I. Angaben zum Kind/ Jugendlichen

**Frage 1: Wie ist die Muttersprache des Kindes oder der/des Jugendlichen?**

**Frage 2: Hat das Kind oder der/die Jugendliche eine Leseschwäche? Kreuzen Sie an.**			
	Ja		Nein

**Frage 3: Gibt es derzeit Probleme/Krisen bei dem Kind/Jugendlichen? Kreuzen Sie an.**			
	Ja		Nein

Falls ja, benennen Sie diese kurz…

II. Angaben zu den Eltern/ Bezugspersonen

	**Frage 4: Hat das Kind psychisch erkrankte Eltern? Kreuzen Sie an.**
	Ja, die Mutter
	Ja, der Vater
	beide Eltern
	andere Bezugspersonen: ____
	Nein

	**Frage 5: Wenn ja, welche psychischen Erkrankungen? (Mehrfachnennung möglich) Kreuzen Sie an.**
	Depression
	Bipolare Störung (Episoden der Manie & Episoden der Depression)
	Angststörungen (z.B. Panikstörung, Agoraphobie, Soziale Phobie, Generalisierte Angststörung)
	Essstörungen (z.B. Magersucht, Bulimie, Binge-Eating-Störung)
	Posttraumatische Belastungsstörung
	Autismus
	Aufmerksamkeits-Defizit-Hyperaktivitäts-Störung
	Zwangsstörungen (z.B. Waschzwang, Durchführen von Ritualen)
	Borderline-Persönlichkeitsstörung
	Schizophrenie oder andere psychotische Erkrankungen
	Suchterkrankungen (z.B. Alkoholsucht, Drogensucht, Spielsucht)
	Demenz

	**Frage 6: Hat das Kind oder der/die Jugendliche regelmäßigen Kontakt zu den Eltern? Kreuzen Sie an.**		
	Ja		Nein

**Falls ja, wie oft steht das Kind oder der/die Jugendliche mit den Eltern in Kontakt? Kreuzen Sie an.**	
	Jeden Tag
	Mehrmals in der Woche
	1x pro Woche
	Mehrmals im Monat
	1x pro Monat
	Ein paar Mal im Jahr

**Frage 7: Wie lange ist das Kind oder der/ die Jugendliche schon in der Einrichtung? Kreuzen Sie an.**	
	Weniger als 1 Jahr
	1 bis unter 2 Jahren
	2 bis unter 3 Jahren
	3 bis unter 6 Jahren
	Über 6 Jahre

**Frage 8: Wie lange ist das Kind oder der/ die Jugendliche schon am Jugendamt angeschlossen? Kreuzen Sie an.**	
	Weniger als 1 Jahr
	1 bis unter 2 Jahren
	2 bis unter 3 Jahren
	3 bis unter 6 Jahren
	Über 6 Jahre

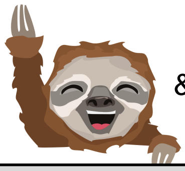

Frage 9: Haben die Eltern einen Migrationshintergrund? Kreuzen Sie an.			
	Ja		Nein

Vielen lieben Dank!
