# Supplementary material for: A Creative and Movement-Based Blended Intervention for Children in Outpatient Residential Care: A Mixed-Method, Multi-Center, Single-Arm Feasibility Trial
Source: Children (Basel). 2023 Jan 24;10(2):207. doi: 10.3390/children10020207 (PMC9954900; doi:10.3390/children10020207)
Supplement: Supplementary file 1 [file children-10-00207-s001.zip › Supplementary material_BirnkammerCalvano/S4.pdf]

& Action, Datum: ________
Dein Code: ___________

Interviewleitfaden & Action ist ein Projekt der YIN YOUNG & YOU gUG
(haftungsbeschränkt) Ausgründung der Freien Universität Berlin

Projektlaufzeit: 01.01.2022 - 31.08.2022

YIN YOUNG & YOU
Mondori App

Titel der Studie: & Action! The effectiveness of a creative blended care intervention for children in residential care: Results of a single arm, multicenter pilot study

Post Testung der Mitglieder der Institution

Susanne Birnkammer, Wissenschaftliche Mitarbeiterin am Fachbereich Erziehungswissenschaften und Psychologie, Arbeitsbereich Klinische Kinder- und Jugendpsychologie und -psychotherapie, Habelschwerdter Allee 45, 14195 Berlin, E-Mail: susanne.birnkammer@fu-berlin.de

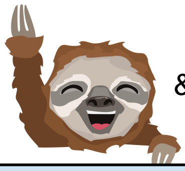

& Action, Datum: _________
Dein Code: ____________

I. Einleitung Post- Testung der Mitglieder

➜ **Gesprächsbeginn:**

Liebe Mitglieder der Institution,
wir hoffen, unser Projekt in Ihrer Einrichtung hat Ihnen gefallen! Als nächstes möchten wir Ihnen einige Fragen diesbezüglich stellen. Falls Sie Fragen oder Anmerkungen haben, können Sie diese gerne im Nachgang stellen. Ihr Feedback hilft uns bei der Verbesserung der App/des Programms, weswegen wir Sie bitten, die Fragen so ausführlich wie möglich zu beantworten. Wir teilen die Fragen auf in Fragen zur App Mondori, der Präsenzübungen sowie dem Projekt im Ganzen.
Beginnen wir mit der ersten Frage.

II. Angaben zur App

➜ **Um die App besser zu gestalten, hätten wir jetzt noch einige Fragen an Sie.**

**Frage 1: Was fanden Sie an der Mondori App besonders gut/effektiv?**

➜ **Danke für dein Feedback zu der App.**

**Frage 2: Was hat Ihnen an der Mondori App nicht gefallen?**

➜**Vielen Dank.**

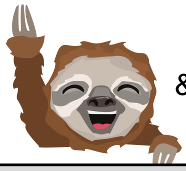

& Action, Datum: ________
Dein Code: ____________

Frage 3: Welche Schulnote würden Sie der App vergeben?

Frage 4:Wie häufig wurde die App von den Kindern/Jugendlichen verwendet?

Frage 5: Was denken Sie, wie die App den Kindern/Jugendlichen gefallen hat?

Frage 6: Welchen Eindruck haben Sie, bezüglich der Weiternutzung der App bei den Kindern/Jugendlichen?

Frage 7: Wie hat Ihnen das Design der App gefallen? Kreuzen Sie an.	
a	Mir hat es überhaupt nicht gefallen
b	Mir hat es nicht so gut gefallen
c	Ich fand es weder gut noch schlecht
d	Mir hat es gut gefallen
e	Mir hat es sehr gut gefallen

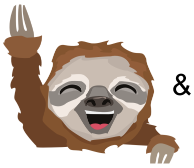

Frage 8: Wie würden Sie insgesamt Ihre Erfahrung mit dem &Action Programm und der Mondori App bewerten? Kreuzen Sie an.	
Notizen:	
a	Mir hat es überhaupt nicht gefallen
b	Mir hat es nicht so gut gefallen
c	Ich fand es weder gut noch schlecht
d	Mir hat beides gut gefallen
e	Mir hat beides sehr gut gefallen

Frage 9: Würden Sie unsere App Mondori anderen weiterempfehlen? Kreuzen Sie an.	
a	Ja, ich würde die Mondori App anderen weiterempfehlen!
b	Nein, ich würde die Mondori App anderen nicht weiterempfehlen!

➜ **Wenn nein, warum würden Sie die App nicht weiter empfehlen?**
(Notizen):

____

____

____

III. Angaben zu Präsenzübungen

➜ **Jetzt möchten wir Ihnen noch einige Fragen zu den therapeutischen Übungen vor Ort stellen**

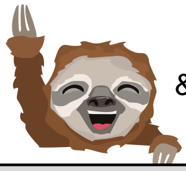

& Action, Datum: _________
Dein Code: ____________

**Frage 10: Welche Therapieart, denken Sie, hat den Kindern/Jugendlichen am meisten Spaß gemacht?**

**Frage 11: Welche Therapieart, denken Sie, waren für die Kinder/ Jugendlichen schwieriger zu bewältigen? Falls ja, durch welche Barrieren gekennzeichnet?**

**Frage 12: Welche Therapieart, denken Sie, waren für die Kinder/ Jugendlichen schwieriger zu bewältigen? Falls ja, durch welche Barrieren gekennzeichnet?**

IV. Angaben zum Projekt

**Frage 13: Hättest du dir eine längere Laufzeit des Projektes (App mit 12 Inseln, Präsenzübungen) gewünscht? (12 Wochen)? Kreuzen Sie an.**	
	Ja
	Nein
	Vielleicht, wenn folgendes anders wäre: ______________________________

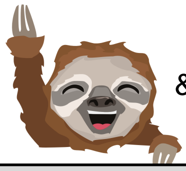

**Frage 14: Was hat Ihnen generell an das Projekt gefallen?**

**Frage 15: Welches der beiden Angebote, hat Ihnen besser gefallen? Kreuzen Sie an.**	
	Präsenzübung
	Mondori App
	Falls ja, warum?

➜ Falls Sie weitere Anmerkungen haben, freuen wir uns sehr! Entweder schreiben Sie diese direkt hier auf oder Sie erreichen uns auch jederzeit unter der Email Adresse info@ying-young-you.com!

Anmerkungen/Fragen der interviewten Person:
____
____
____
____

V. Gesprächsabschluss

➜ Damit wären wir am Ende unseres Interviews. Wir bedanken uns herzlichst für die Beantwortung der Fragen und die Testung unserer App Mondori zusammen mit den Kindern/Jugendlichen! Wir freuen uns, wenn Sie Mondori auch zukünftig verwenden!

Ihr Team von &Action
